# Impact of Social Isolation on the Behavioral, Functional Profiles, and Hippocampal Atrophy Asymmetry in Dementia in Times of Coronavirus Pandemic (COVID-19): A Translational Neuroscience Approach

**DOI:** 10.3389/fpsyt.2020.572583

**Published:** 2020-11-24

**Authors:** Aida Muntsant, Lydia Giménez-Llort

**Affiliations:** ^1^Department of Psychiatry and Forensic Medicine, School of Medicine, Universitat Autònoma de Barcelona, Barcelona, Spain; ^2^Institut de Neurociències, Universitat Autònoma de Barcelona, Barcelona, Spain

**Keywords:** isolation, confinement, risk factors, neuropsychiatric symptoms in dementia, gender, nursery homes, animal models-rodent, brain asymmetry

## Abstract

The impact of COVID-19 on the elderly is devastating, and nursing homes are struggling to provide the best care to the most fragile. The urgency and severity of the pandemic forces the use of segregation in restricted areas and confinement in individual rooms as desperate strategies to avoid the spread of disease and the worst-case scenario of becoming a deadly trap. The conceptualization of the post–COVID-19 era implies strong efforts to redesign all living conditions, care/rehabilitation interventions, and management of loneliness forced by social distance measures. Recently, a study of gender differences in COVID-19 found that men are more likely to suffer more severe effects of the disease and are over twice as likely to die. It is well-known that dementia is associated with increased mortality, and males have worse survival and deranged neuro-immuno-endocrine systems than females. The present study examines the impact of long-term isolation in male 3xTg-AD mice modeling advanced stages of Alzheimer's disease (AD) and as compared to age-matched counterparts with normal aging. We used a battery of ethological and unconditioned tests resembling several areas in nursing homes. The main findings refer to an exacerbated (two-fold increase) hyperactivity and emergence of bizarre behaviors in isolated 3xTg-AD mice, worrisome results since agitation is a challenge in the clinical management of dementia and an important cause of caregiver burden. This increase was consistently shown in gross (activity in most of the tests) and fine (thermoregulatory nesting) motor functions. Isolated animals also exhibited re-structured anxiety-like patterns and coping-with-stress strategies. Bodyweight and kidney weight loss were found in AD-phenotypes and increased by isolation. Spleen weight loss was isolation dependent. Hippocampal tau pathology was not modified, but asymmetric atrophy of the hippocampus, recently described in human patients with dementia and modeled here for the first time in an animal model of AD, was found to increase with isolation. Overall, the results show awareness of the impact of isolation in elderly patients with dementia, offering some guidance from translational neuroscience in these times of coronavirus and post–COVID-19 pandemic. They also highlight the relevance of personalized-based interventions tailored to the heterogeneous and complex clinical profile of the individuals with dementia and to consider the implications on caregiver burden.

## Introduction

The impact of COVID-19 on the elderly is devastating despite nursing homes and residential care homes struggling to provide the best care to the most fragile of the elderly population, who due to their physical and/or mental conditions already needed continuous surveillance and/or professional care. Between 70 and 80% of nursing home residents are affected by dementia (1), a mental disease that, *per se*, is associated with increased mortality in comparison with aged control populations (2, 3). In these scenarios, the severe constraints that all other parts of the society are facing not only exist but also may have a greater impact on the elderly due to their vulnerability, frailty, and increased risk of mortality (4).

Official data on the numbers of death linked to COVID-19 in care home residents are still difficult to ascertain. Differences in testing and the policies of different countries make international comparisons difficult. Despite these difficulties, it is obvious that nursing home residents have a higher risk of dying from COVID-19. The most recent report in Europe (Amsterdam) shows that mortality is higher in residents with confirmed COVID-19 with more typical symptoms (fever, dyspnea, coughing), reaching 37%, but even when no typical symptoms but sore throat and delirium are present, mortality in residents with confirmed COVID-19 is 27% (5). Early international evidence, with the actual official data, indicates that about half of all people who have died from COVID-19 were care home residents (6).

The urgency and severity of this pandemic has forced care institutions to use segregation in restricted areas (floors) and confinement in individual rooms as desperate strategies, first, to avoid the spread of disease, and thereafter to hamper the worst-case scenario of becoming a deadly trap. With visits to nursing homes and long-term care facilities banned, residents have no face-to-face contact with their families. Group activities are no longer possible in care homes or nursing homes. Residents have become more socially isolated, lacking the social and environmental enrichment that are key rehabilitation factors to counteract their progressive physical and/or mental deterioration. Moreover, redistribution and relocation of rooms can increase the stress and behavioral problems as the new arrangements become a new environment for the patient (7, 8).

While we wait for the vaccine to arrive, the conceptualization of elderly care in the post–COVID-19 era implies strong efforts to redesign all living conditions and lifestyles, to find new care and rehabilitation strategies, and to provide better management of loneliness forced by physical distance. Due to the impact of isolation on mental well-being, the WHO cautions about the incorrect use of the term “social distancing” to refer to a number of measures to increase physical space between people to prevent the spread of COVID-19 (9). It also highlights the relevance of referring instead to “physical distancing” and to conceptualize the policies to avoid disconnection from family and loved ones because in the current situation there is a pressing need to stay emotionally and socially connected (9). Social vulnerability, frailty, and mortality are a dangerous triad for the elderly as well (4). Social isolation is considered a risk factor for morbidity and mortality since it strongly contributes to comorbid conditions such as hypertension, cardiovascular disease, cognitive decline, depression, and early mortality or the disabilities related to them (10–14). Social isolation not only increases the risk of dementia but it is also associated with an increased memory decline and exacerbation of the symptoms in dementia patients (15, 16). Thus, the singular features of this pandemic will also generate collateral effects in people's health, mostly in patients (those already ill) without COVID-19 (17). Recent reports suggest that a pandemic can be a traumatic experience for some individuals and lead to posttraumatic stress disorder (PTSD). Although it may depend on several other risk factors, the COVID-19 pandemic can be a trigger for mental health problems (18, 19). The emergence of psychiatric problems and/or deterioration of mental health are getting attention, and technical guides for interventions focusing on the individual needs are being developed urgently. New adaptations, such as telecare, are playing a very important role (20, 21). Telepsychiatry is becoming a useful tool for patients with mental disorders (22). For example, a current report on older adults with mild cognitive impairment or mild dementia performed by Telehealth home support shows that those who were living alone during the confinement reported decreased well-being, greater anxiety, and more sleeping problems (23). Coping with stress and negative and sad emotions have a stronger impact on the elderly due to their age-related immunosenescence and the neuro-immune dysregulation associated with these psycho-social processes (24).

A current study of gender differences in COVID-19 found that in spite of males and females having the same prevalence of the disease, males are more at risk for worse outcomes and death independent of age (25). According to the morbidity/mortality paradox, also in dementia males show worse survival and deranged neuro-immuno-endocrine system than females despite their less bad neuropathological status (2, 3). AD, as many other forms of dementia, is one of the principal causes of disability in old adulthood. It is defined as an accumulation of amyloid-β (Aβ) plaques and tau-containing neurofibrillary tangles (NFTs), accompanied by a progressive memory decline (26). Neuropsychiatric symptoms (NPS) (27), also called behavioral and psychological symptoms of dementia (BPSD), are observed in 90% of patients and may include agitation, anxiety, verbal or physical aggression, sundowning behavior, wandering, depression, challenging and disruptive behaviors, hallucinations, etc. For instance, the prevalence of aggression is between 33 and 46%, with males especially affected (28, 29). These NPS are highly associated with caregiver burnout (30), frequently leading to institutionalization (31) as their management usually needs pharmacological treatment supported by non-pharmacological interventions. However, despite efficacy in the reduction of most of these challenging symptoms by antipsychotics (32), they should not be used routinely for the treatment of aggression and psychosis in these patients because they have an associated increased risk of cerebrovascular accidents, respiratory diseases, and mortality (33–35). Unfortunately, at a practical level, the medication reviews are infrequent, and antipsychotics are used on a long-term basis despite their higher mortality risk (36). It also has been noted that care homes usually present a higher prevalence of antipsychotic prescription, and this seems to be increased in those with fewer staff (37, 38), important data to be taken into account within the pandemic consequences.

The present report aims to address these questions from a translational approach, studying the impact of long-term isolation in male 3xTg-AD mice modeling advanced stages of AD and as compared to age-matched group-housed 3xTg-AD mice and non-transgenic (NTg) counterparts with normal aging. This mice model of AD, created in the laboratory of Prof. Frank M. LaFerla at the University of California, Irvine, harbors PS1/M146V, APPswe, and tau P301L human transgenes and uniquely mimics various symptoms of the disease in temporal and neuroanatomical patterns like those observed in patients (39, 40). Moreover, 3xTg-AD male mice have shown increased mortality as compared to females and NTg counterparts (41–44), which means many 3xTg-AD males arrived at old age in socially isolated housing conditions. The impact of individual housing conditions at different ages of the rodents' lifespan is used as an animal model for some mental health disorders. Post-weaning social isolation of mice is a well-established animal model for developmental disorders, such as attention-deficit hyperactivity disorder, autism spectrum disorder, and specific learning disability (45). In the case of adolescent rodents, social isolation is regarded as a model of heightened vulnerability to comorbid alcoholism and anxiety disorders (46), while in adults it is used for pharmacological treatments for PTSD (47). Yet, only a few laboratories have studied the effects of poor social housing, and they show a worsening of the cognitive and beta-amyloid pathology in animal models for dementia (48–50). Here, for the first time, we investigated the impact of long-term social isolation in the elder 3xTg-AD male mice, focusing on several cognitive, BPSD-like, and daily-life-activity functions, physical status (body, liver, kidney, and spleen weights) as well as the neuropathological AD-phenotype. Thus, the animals were confronted with a battery of tests that included enclosures of differing levels of anxiogenic conditions that, for the purpose of the present work, could resemble or translate into several areas in nursing homes.

## Materials and Methods

### Animals

A total of 29 11-month-old (11.1 ± 0.16) male mice from the Spanish colonies of homozygous 3xTg-AD (*n* = 20) and non-transgenic (NTg, *n* = 9) mice in a C57BL/6 background were used. The 3xTg-AD mice were genetically engineered at the University of California, Irvine as described previously (51).

Animals were housed three or four per cage or isolated and maintained in Macrolon cages (35 × 35 × 25 cm) under standard laboratory conditions of food and water ad libitum, 22 ± 2°C, a 12-h light/dark cycle, and relative humidity of 50–60%. When isolated, animals still were socially connected through olfaction and audition, and as in the group-housed conditions, the cages were enriched with nesting materials. Behavioral assessments were performed blind to the experiment, in a counterbalanced manner, in the light cycle, from 09:00 to 13:00 h. All procedures were in accordance with the Spanish legislation on the “Protection of Animals Used for Experimental and Other Scientific Purposes” and the EU Directive (2010/63/UE) on this subject. The protocol CEEAH 3588/DMAH 9452 was approved by Departament de Medi Ambient i Habitatge, Generalitat de Catalunya. The study complies with the ARRIVE guidelines developed by the NC3Rs and the aim to reduce the number of animals used.

### Behavioral Assessments

The impact of social isolation was measured in 11-month-old 3xTg-AD mice after a long-term, naturally occurring housed isolation (8.5 ± 0.3 months). The battery of behavioral tests consisted of a series of classical unconditioned tasks measuring locomotion and exploratory activity, anxiety-like behaviors, cognitive functions, and daily life activities (39). The results were compared to age-matched, group-housed 3xTg-AD mice and non-transgenic counterparts with normal aging.

#### Day 1: Corner Test and Open Field Test

Neophobia to a new home-cage was assessed by a 30-s trial. Animals were placed individually in the center of a clean standard home-cage, filled with wood shaving bedding. The number of corners visited was recorded (52). The latency to realize the first rearing (vertical displacement) and the number of rearings were also registered (39).

Immediately after the corner test, mice were placed in the center of an illuminated (20 lx) open field (metalwork, white box, 42 × 38 × 15 cm) and exploratory and anxiety-like behaviors were evaluated during 5 min (53). First, the ethogram of action programs described by the temporal profile of the following sequence of behavioral events was recorded: duration of freezing behavior, latency to leave the central square and enter the peripheral ring, and performing first wall rearing as well as latency and total duration of self-grooming behavior. Second, the time course and total levels of exploratory activity were measured as horizontal (number of crossings of 10 × 10-cm squares) and vertical (rearings with a wall support) locomotor activity. Third, variables of emotionality (54) included the number of defecations, the presence of urine, and the grooming behavior, their number, latency, and total time. Finally, as described previously (55), we evaluated the presence of bizarre behaviors such as stereotyped rearings without wall support.

#### Day 2: Spontaneous Activity Test

The mice were individually tested in a new home-cage containing a small amount (25 ml) of clean sawdust on the floor. Here, (horizontal) spontaneous locomotor activity was continuously recorded during a 45-min period and short-term spatial habituation was determined by means of the VideoTrack analysis system (ViewPoint Behavior Technology, Lion, France) (56).

#### Day 3: T-Maze

Coping-with-stress strategies, risk assessment, and working memory were assessed in a spontaneous alternation task (57) in a black T-shaped maze. The apparatus consisted of a woodwork, two short arms of 30 × 10 cm, and a long arm of 50 × 10 cm. The animal was placed inside the long arm of the maze with its head facing the end wall, and it was allowed to explore the maze for a maximum of 5 min. The goal latencies, namely to move and turn (freezing behavior), to reach the intersection, the time elapsed until the animal crossed (4 paws criteria) the intersection of the three arms, and the total time invested to explore the three arms of the maze (test completion criteria) were recorded. The entry of an already visited arm in the trial before completing the test was considered an error. Defecation boli and urination were also noted.

#### Days 4–6: Nesting

In a regular manner, for animal well-being, the cages were enriched with nesting material. However, in order to specifically assess this daily life activity, our 3-day protocol for nesting behavior using tissue paper was used (58). One paper towel tissue (70 × 30 mm of “paper towel”) (Sudelab, Rubí, Barcelona, Spain) was introduced in the home-cage. On the next day, 48 and 72 h later, the nests were assessed according to the Deacon 5-point scale (59) from 1 to 5 as follows: 1 = not noticeably touched, 2 = partially torn up, 3 = mostly shredded but often no identifiable site, 4 = identifiable but flat, 5 = perfect or nearby. Pictures were taken prior to evaluation for documentation.

#### Day 6: Pathological Status

Bodyweight was recorded to monitor the physical status of animals. The size (weight in mg) and relative size (% vs. bodyweight) of liver, kidneys, and spleen were also recorded, as an indirect measure of the physiological status of the metabolic, excretory, and peripheral immune organs, respectively (41). Tissue samples were stored at −80°C for further biochemical analysis (see below). For biochemical analysis of the level of tau pathology, right and left hippocampus were dissected, frozen in dry ice, and kept at −80°C. Thereafter, frozen samples were lysed in cold lysis buffer containing protease and phosphatase inhibitors (Sigma). Protein content was quantified with the BCA Protein Assay Kit (Thermo Scientific), resolved with SDS-polyacrylamide gel electrophoresis, and detected by Western blotting using the mouse anti-phosphorylated Ser396/404 (PHF-1; 1:1000) antibody. Quantification was performed using the ImageStudio Lite 5.2 software.

### Statistics

Results are expressed as mean ± SEM. SPSS 20.0 software was used. Differences were studied using multivariate general linear model analysis, followed by *post hoc* Duncan's tests when possible. Repeated measures ANOVA was used to study differences in the time course of behaviors. Student's *t*-test was used to compare two independent groups, per genotype or isolation. For categorical variables, chi square or Fisher's exact test with 2 × 2 were used. Pearson's correlation analysis evaluated the pathological/behavioral correlates. In all the tests, *p* < 0.05 was considered statistically significant.

## Results

**Figure 1:**
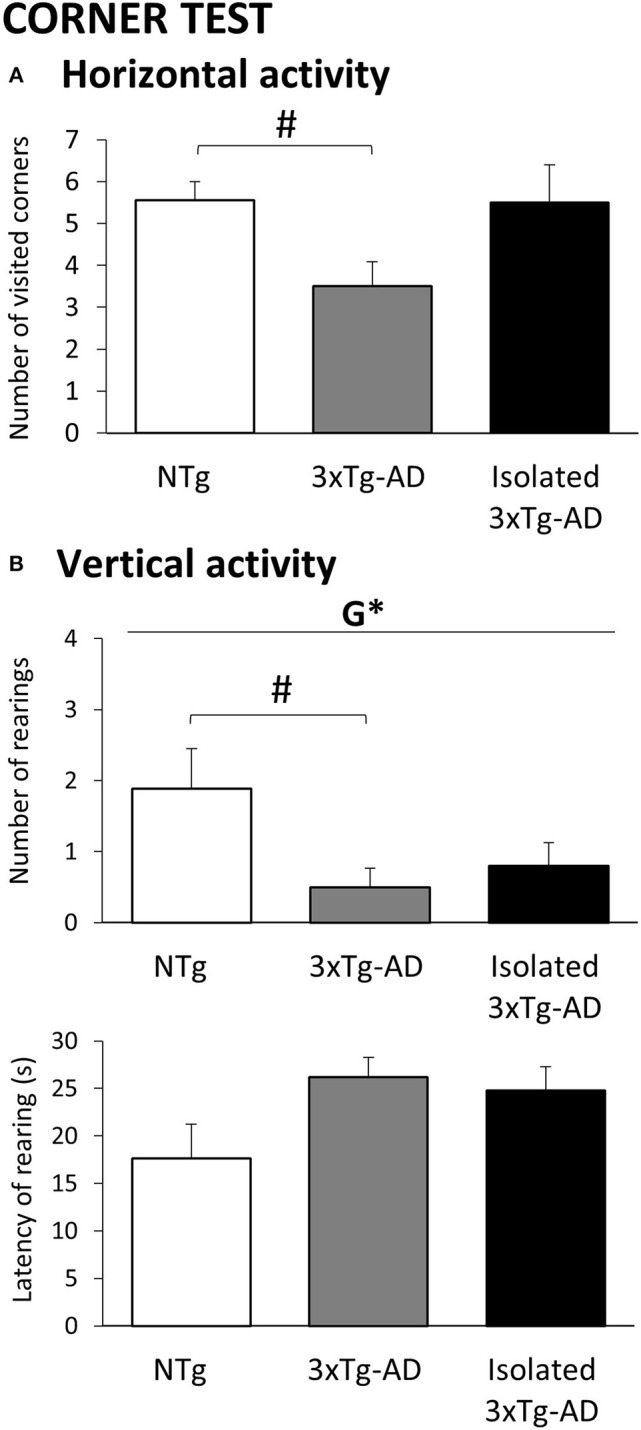
Effects of isolation on 11-month-old male 3xTg-AD mice in the Corner test. Results are expressed as mean ± SEM. **(A)** Horizontal activity; **(B)** Vertical activity. Groups: NTg, group-housed non-transgenic mice; 3xTg-AD, group-housed 3xTg-AD mice; Isolated 3xTg-AD, single-housed 3xTg-AD mice. Statistics: G, genotype effect, *p < 0.05. Student *t*-test comparisons are shown in the graphs as ^#^*p* < 0.05 vs. the NTg-group.

**Figure 2:**
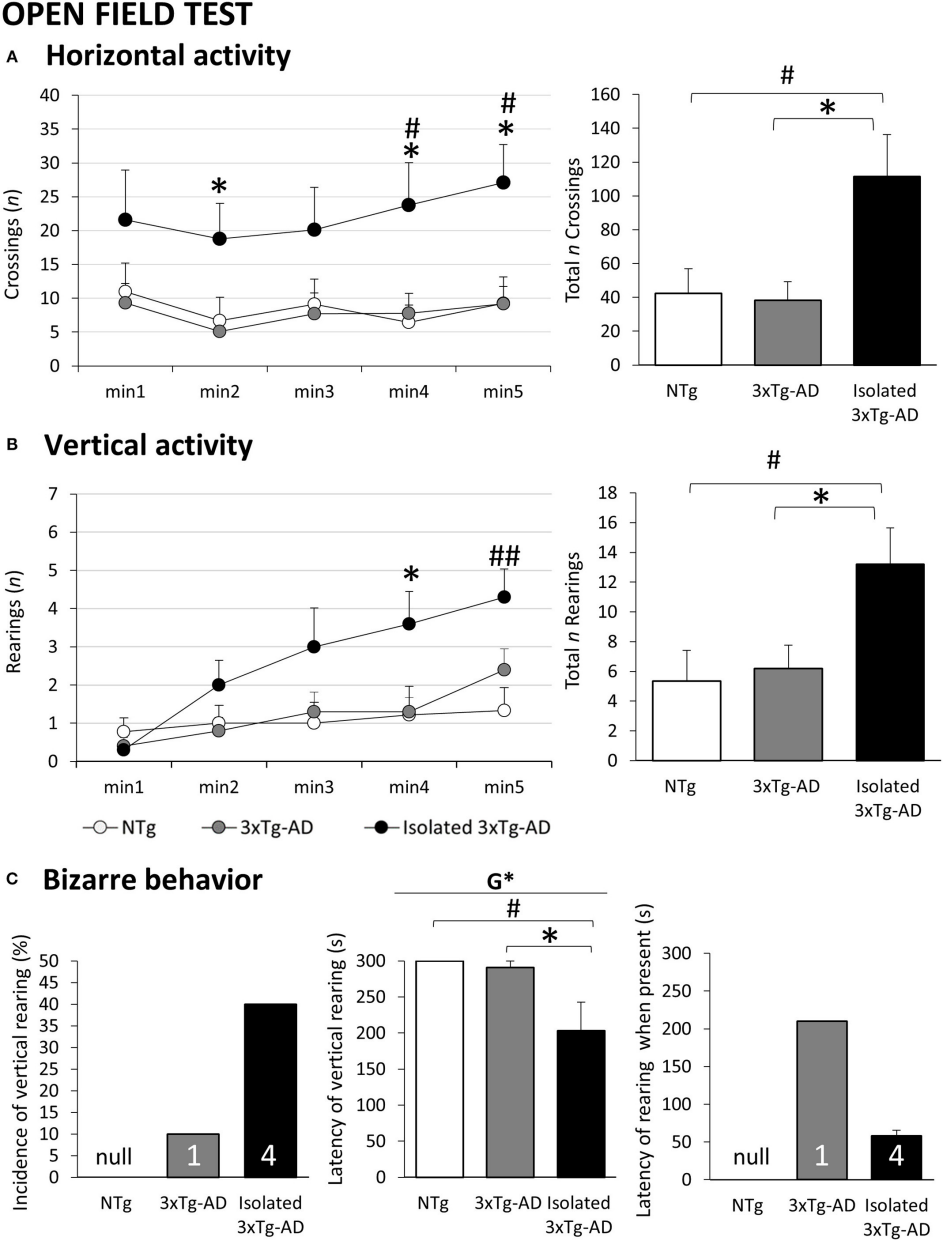
Effects of isolation on 11-month-old male 3xTg-AD mice in the Open field test. Results are expressed as mean ± SEM or incidence (%). **(A)** Horizontal activity: Left, time course; right, total; **(B)** Vertical activity: Left, time course; right, total; **(C)** Bizarre behavior: Incidence (left), latency (center), latency when present. Groups: NTg, group-housed non-transgenic mice; 3×Tg-AD, group-housed 3xTg-AD mice; Isolated 3xTg-AD, single-housed 3xTg-AD mice. Inset of bars, number of animals exhibiting the behavior. Statistics: G, genotype effect, *p < 0.05. Student *t*-test comparisons are shown as ^#^*p* < 0.05, ^##^*p* < 0.01 vs. the NTg-group; *p < 0.05 vs. the 3×Tg-AD mice.

**Figure 3:**
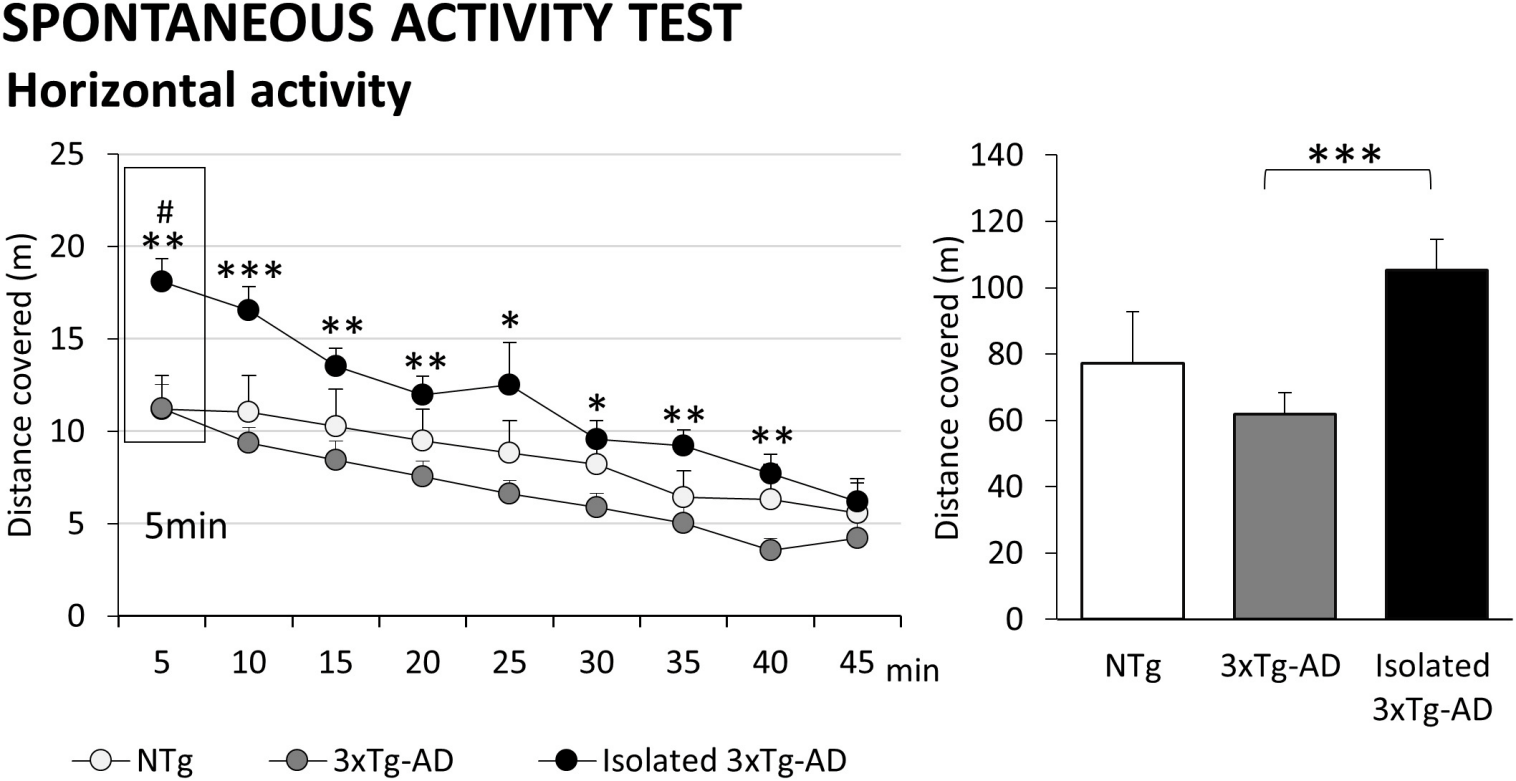
Effects of isolation on 11-month-old male 3xTg-AD mice in the Spontaneous activity test. Results are expressed as mean ± SEM. Horizontal activity: left, time course; right, total distance covered. Groups: NTg, group-housed non-transgenic mice; 3xTg-AD, group-housed 3xTg-AD mice; Isolated 3xTg-AD, single-housed 3xTg-AD mice. Statistics: Student *t*-test comparisons are shown as ^#^*p* < 0.05 and vs. the NTg-group; *p < 0.05, **p < 0.01, ***p < 0.001 vs. the 3xTg-AD mice.

**Figure 4:**
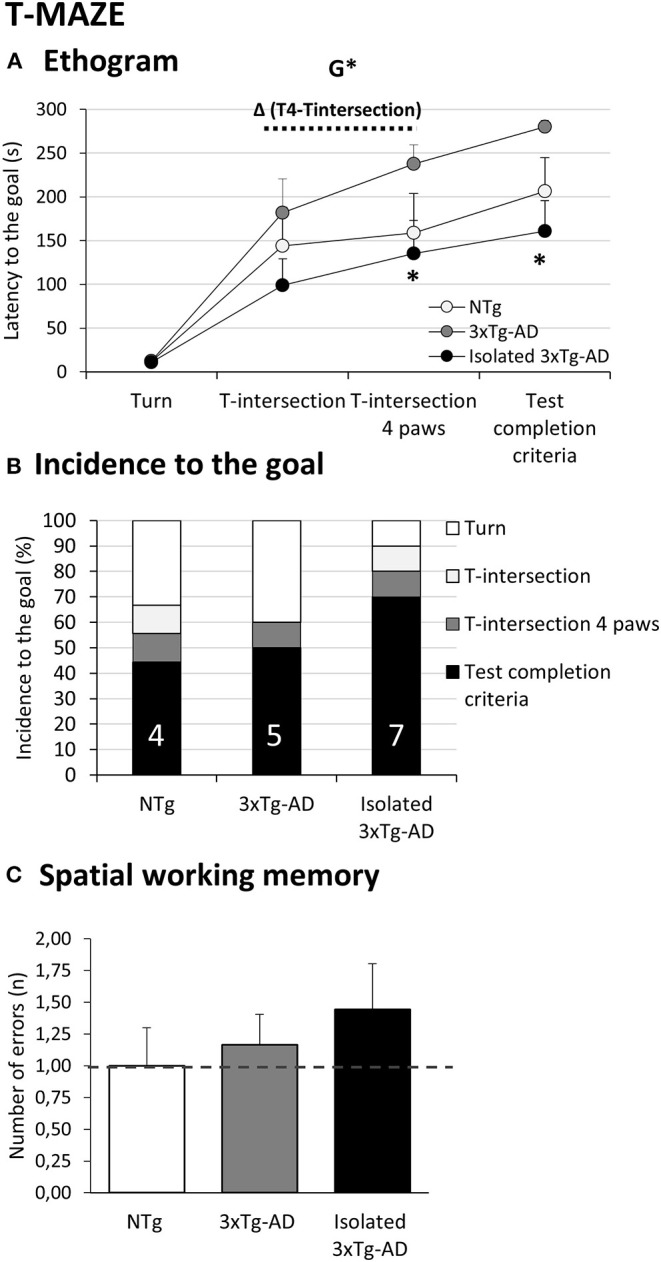
Effects of isolation on 11-month-old male 3xTg-AD mice in the T-maze. Data are expressed as the mean ± SEM or incidence (%). **(A)** Ethogram; **(B)** Incidence to the goal; **(C)** Spatial working memory. Groups: NTg, group-housed non-transgenic mice; 3xTg-AD, group-housed 3xTg-AD mice; Isolated 3xTg-AD, single-housed 3xTg-AD mice. Inset of bars, number of animals exhibiting the behavior. Statistics: G, genotype effect, *p < 0.05. Student *t*-test comparisons are shown as *p < 0.05 vs. the 3xTg-AD mice.

**Figure 5:**
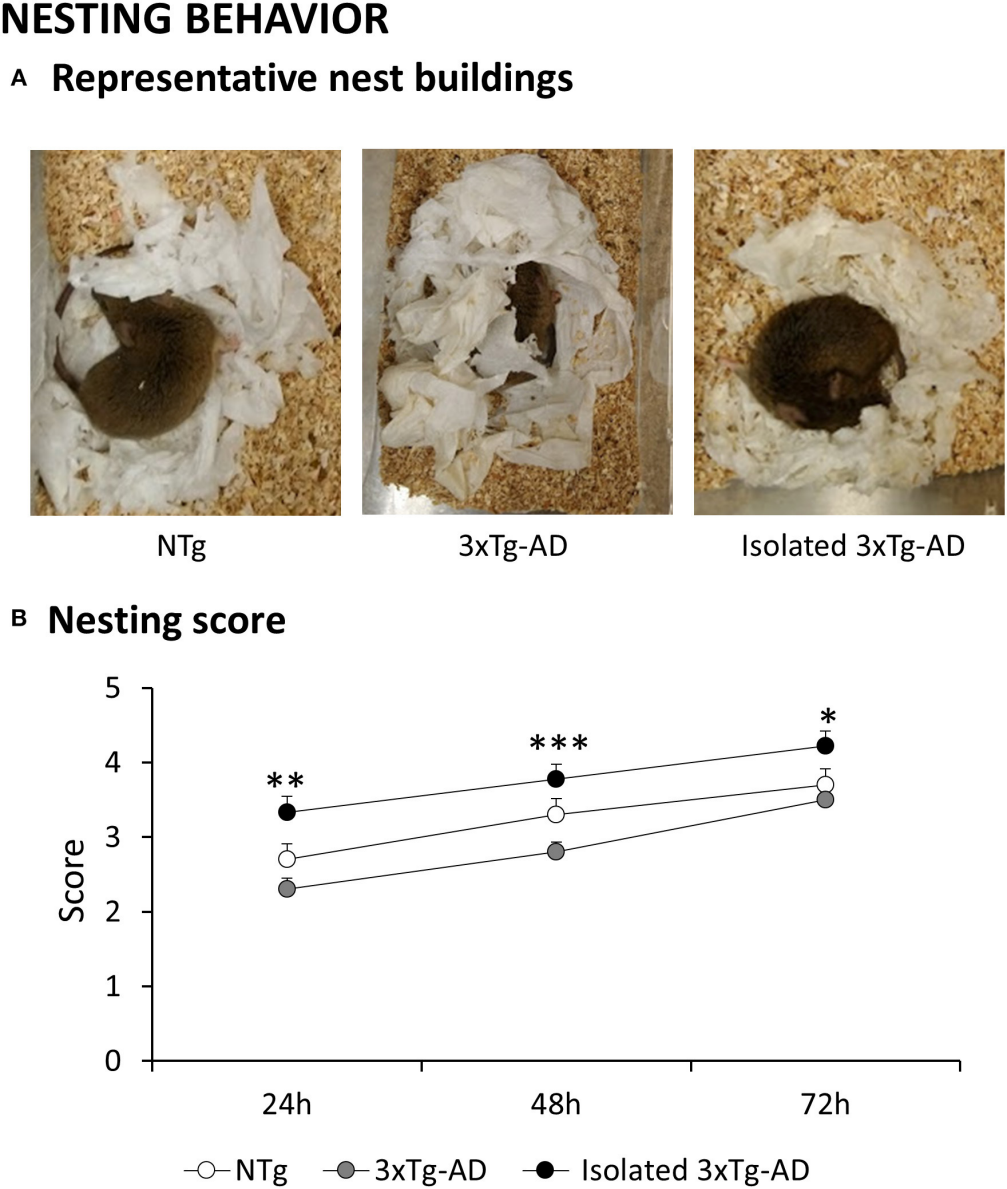
Effects of isolation on 11-month-old male 3xTg-AD mice in the Nesting behavior. Data are expressed as the mean ± SEM. **(A)** Representative images of nest buildings: **(B)** Nesting score at 24, 48, and 72 h. Groups: NTg, group-housed non-transgenic mice; 3xTg-AD, group-housed 3xTg-AD mice; Isolated 3xTg-AD, single-housed 3xTg-AD mice. Statistics: Student *t*-test comparisons are shown as *p < 0.05, **p < 0.01, ***p < 0.001 vs. the 3xTg-AD mice.

**Figure 6:**
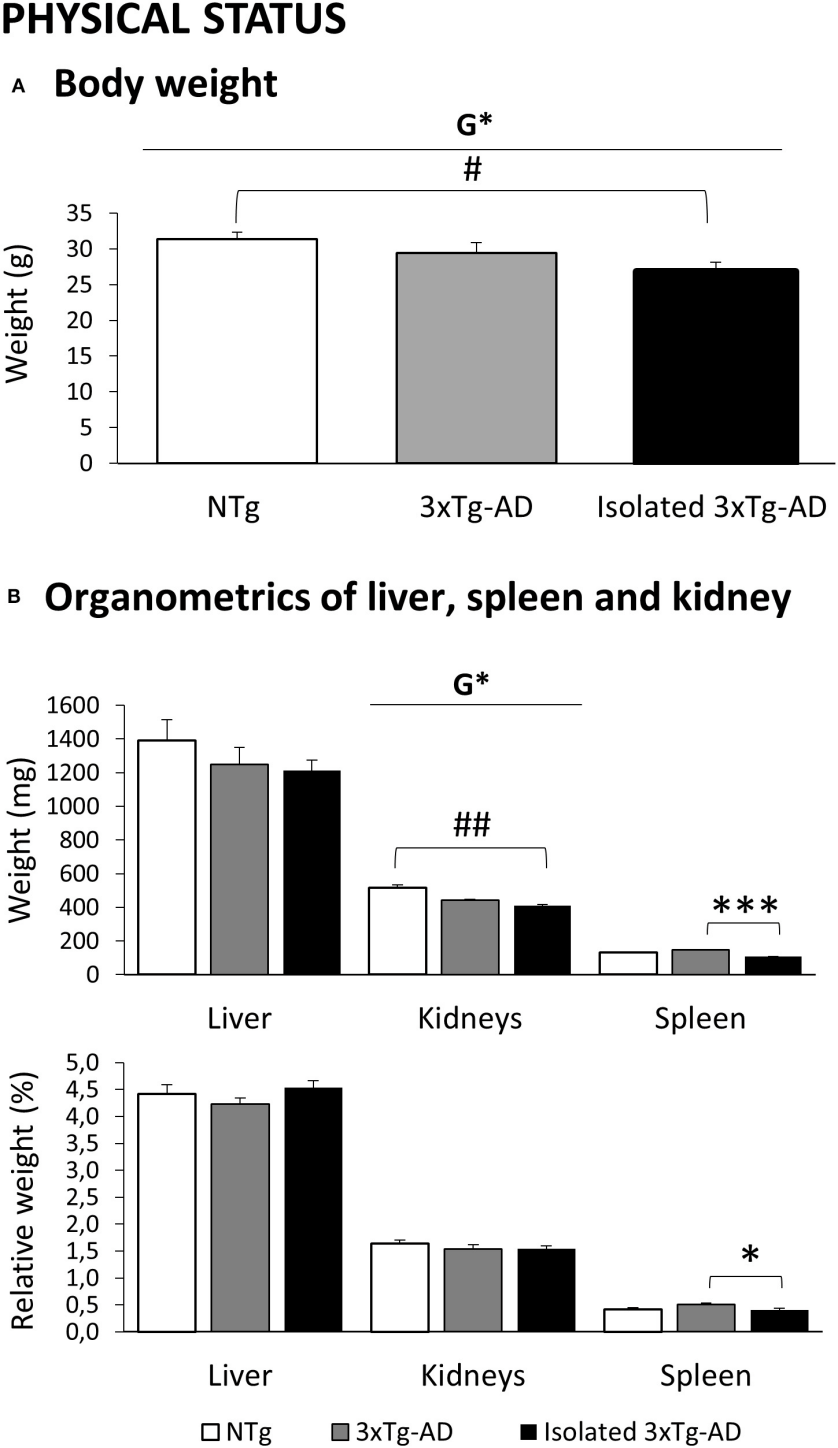
Effects of isolation on 11-month-old male 3xTg-AD mice in the Physical status. Results are expressed as mean ± SEM. **(A)** Body weight; **(B)** Organometrics of liver, spleen, and kidney. Groups: NTg, group-housed non-transgenic mice; 3xTg-AD, group-housed 3xTg-AD mice; Isolated 3xTg-AD, single-housed 3xTg-AD mice. Statistics: G, genotype effect, *p < 0.05. Student *t*-test comparisons are shown as ^#^*p* < 0.05, ^##^*p* < 0.01 vs. the NTg-group; *p < 0.05, ***p < 0.001 vs. the 3xTg-AD mice.

**Figure 7:**
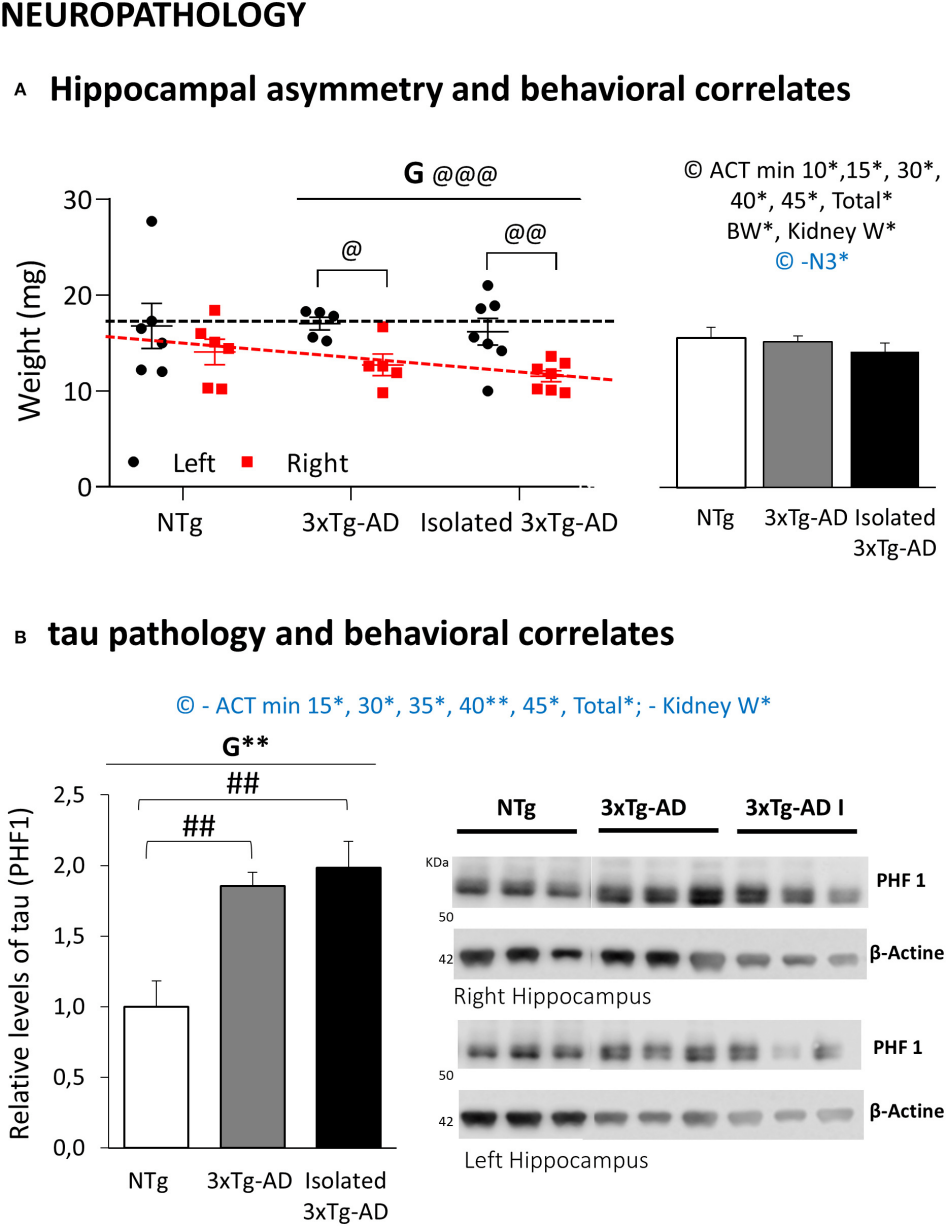
Effects of isolation on 11-month-old male 3xTg-AD mice in Neuropathology, R/L hippocampal asymmetry, and Behavioral correlates. Data are depicted and also expressed as mean ± SEM. **(A)** Hippocampal asymmetry; Left, R/L weights, Individual data of the corresponding area in the left (black) and right (red) hemispheres; Right, mean hippocampal weight and behavioral correlates. **(B)** Tau pathology; Left, Relative levels of tau (PHF1); Right, Western blots of right and left hippocampus. Groups: NTg, group-housed non-transgenic mice; 3xTg-AD, group-housed 3xTg-AD mice; isolated 3xTg-AD, single-housed 3xTg-AD mice. Statistics: Paired *t*-test: G, genotype effect in asymmetry between R/L hippocampus, ^@@@^*p* < 0.001. Paired *t*-test R/L asymmetry in each group; ^@^*p* < 0.05, ^@@^*p* < 0.01. G, genotype effect, **p < 0.05. Student *t*-test comparisons are shown as ^##^*p* < 0.01 vs. the NTg-group. Positive (black) and negative (blue) Pearson's correlation analysis evaluating hippocampus weight or the hippocampal relative levels of tau/behavioral correlates *p < 0.05; ACT min x, Activity test in the minute x; Total, Total activity in the activity test; BW, Bodyweight; Kidney W, Kidney weight.

The results, depicted in Figures 1–**7** and Table 1, show the impact of social isolation on the behavioral, functional, and neuropathological phenotype of 11-month-old male 3xTg-AD mice, an age mimicking advanced stages of the disease and as compared to age-matched NTg mice.

**Table 1:** Effects of isolation on 11-months-old male 3xTg-AD mice in the Open field test.

**Males, 11-month-old Ethogram and Emotionality Open field test**	**NTg (*n* = 9)**	**3xTg-AD mice** ***n*** **= 20**		**Statistics**	
		**3xTg-AD (*n* = 10)**	**Isolated 3xTg-AD (*n* = 10)**	**AD Genotype**	**Isolation**
Initial freezing (latency, s)	16.44 ± 12.40	17.80 ± 6.08	9.00 ± 2.61	n.s	n.s
Exit of the center (latency, s)	45.22 ± 19.39	59.30 ± 31.08	22.30 ± 12.65	n.s	n.s
Entrance to the periphery (latency, s)	68.67 ± 33.11	60.60 ± 30.96	64.00 ± 28.66	n.s	n.s
Vertical activity (latency, s)	146.89 ± 41.68	131.90 ± 34.67	116.40 ± 27.22	n.s	n.s
Thigmotaxis (total crossings periphery/center)	3.20 ± 0.95	3.70 ± 0.88	3.80 ± 1.18	n.s	n.s
Self-grooming (latency, s)	204.00 ± 32.24	199.10 ± 36.21	162.40 ± 22.27	n.s	n.s
Self-grooming (number)	1.00 ± 0.29	1.20 ± 0.20	1.20 ± 0.20	n.s	n.s
Defecation boli (number)	4.22 ± 0.52	3.90 ± 0.58	4.50 ± 0.45	n.s	n.s

*Ethogram, thigmotaxis, self-grooming and defecation. Results are expressed as mean ± SEM. Groups: NTg, group-housed non-transgenic mice; 3xTg-AD, group-housed 3xTg-AD mice; Isolated 3xTg-AD, single-housed 3xTg-AD mice. All n.s., p > 0.05*.

### Corner Test

The horizontal and vertical behaviors in the corner test are illustrated in Figure 1. Increased neophobia as compared to NTg mice was found in 3xTg-AD mice shown as a statistically significant reduction in the number of visited corners and rearings (*p* < 0.05). In the single-housed 3xTg-AD mice, genetic differences with NTg mice were lost as a result of increased horizontal and vertical activities. Although their rearing latencies did not reach statistical significance, in both 3xTg-AD groups, they were indicative of increased neophobia response.

### Open Field Test

Figures 2A–C illustrates the temporal curves (left) and total accumulated counts (right) of horizontal and vertical exploratory activity during the 5 min in the open field test performed immediately after the corner test. The ethogram was developed similarly in the three groups of mice, and the presence of thigmotaxis indicated the preference for peripheral protected areas as compared to the open and central ones (see Table 1). However, the time course of the horizontal (Figure 2A left, *p* < 0.001) and vertical (Figure 2B left, *p* < 0.001) activities also indicated that isolated 3xTg-AD mice exhibited a hyperactive pattern. Thus, a two-fold increase in horizontal activity (groups *p* < 0.05, *post hoc* isolated 3xTg-AD *p* < 0.05 vs. NTg and vs. 3xTg-AD) and increasing elicitation of vertical exploration through the test (groups *p* < 0.05, *post hoc* isolated 3xTg-AD *p* < 0.05 vs. NTg). These enhanced activity levels resulted in statistically significant increased total levels of both horizontal and vertical activities (Figures 2A,B right, respectively; *p* < 0.05 vs. NTg and vs. 3xTg-AD mice). In contrast, the levels of horizontal activity were very low in both middle-aged NTg and 3xTg-AD mice housed in standard conditions, and their patterns were regular, with poor habituation to the test. As illustrated in Figure 2C, the presence of stereotyped vertical rearing without wall support, absent in NTg mice, was late (210 s), exhibited in only 1/10 group-housed 3xTg-AD mice. In contrast, 4/10 isolated 3xTg-AD mice exhibited this bizarre behavior with an early appearance as part of the fearful response during the first minute of the test (statistics on the mean latency, *p* < 0.05 vs. NTg and vs. 3xTg-AD mice). No statistically significant differences were found among groups in the other variables of the test (see Table 1).

### Spontaneous Activity Test

As illustrated in Figure 3, the spontaneous activity assessed during a long period of 45 min demonstrated a statistically significant sustained hyperactivity pattern induced by isolation in 3xTg-AD mice in contrast to the poor activity levels shown in animals housed in standard conditions. Here, although the locomotor activity levels of 3xTg-AD mice did not reach statistical significance, they were below those of NTg mice. The initial 5-min period reproduced the results found in the 5-min session in the open field test. The hyperactive pattern was evolving during the different periods of habituation to the test, but still with significantly higher levels of activity as compared to 3xTg-AD mice housed in standard conditions (time *p* < 0.001; groups, *p* < 0.05, *post hoc* isolated 3xTg-AD mice vs. 3xTg-AD mice).

### T-Maze

Figure 4A depicts the fast ethogram performed by isolated 3xTg-AD animals as compared to NTg and 3xTg-AD mice in a spontaneous alternation paradigm in the T-maze. Although all animals showed similar latencies to turn from the “facing the wall” starting position, the latency to cross the intersection of the three arms and the latency to complete the task were significantly shorter as compared to the long delays usually exhibited by 3xTg-AD mice (*p* < 0.05 vs. 3xTg-AD). Decision making, measured as the difference between latency to intersection and latency to cross it with 4 paws criteria, was longer in both 3xTg-AD groups, while NTg mice made the decision in a short gap of 10 s (genotype effect, *p* < 0.05). As in the previous tests, the increase in locomotor activity of the isolated 3xTg-AD mice resulted in completing the test faster than the other groups (*p* < 0.05 vs. NTg and vs. 3xTg-AD). As detailed in Figure 4B, a number of animals (3/9) exhibited the NTg coping-with-stress strategy of resting with their backs protected in the starting point. Another two of them spent the time but did not cross the intersection (2/9) or did not complete the test successfully (1/9). Among 3xTg-AD mice, a similar total number of animals did not even start (4/10) or complete (1/10) the test. The proactive strategy of isolated 3xTg-AD mice resulted only in mice resting (1/10), and two were not able to complete the test (2/10). In this small representation (NTg: 6/9; 3xTg-AD: 6/10; isolated 3xTg-AD: 9/10), the number of spatial working memory errors (revisiting an explored area) were recorded in those animals able to initiate the task. Although the sample size was not enough to reach statistical significance, an increased number of errors in spatial alternation was noted in the isolated 3xTg-AD mice (Figure 3C).

### Nesting

Representative nest buildings at 72 h are shown in Figure 5. The effect of isolation was consistently shown since the first day of assessment. While nests of NTg and 3xT-AD mice were partially torn up (mean score 2), those of isolated 3xTg-AD mice were mostly shredded although not yet in an identifiable site (mean score 3). The progressive improvement in nest building allowed us to see a delay in 3xTg-AD mice. Thus, the mean score 3 for nest construction was achieved by NTg at 48 h, but 3xTg-AD mice needed an extra day (72 h). At the endpoint, identifiable but flat nests (mean score 4) were only seen in the isolated group, with nests of very few animals considered perfect or nearly reaching the maximum score of 5 (1/9 NTg, 0/10 3xTg-AD but 3/10 isolated 3xTg-AD mice).

### Pathological Status

Bodyweight of animals was monitored during the battery of behavioral tests as a measure of their physical/healthy status. Figures 6, 7 illustrate the results of a representative sample of animals used for pathological evaluation. As detailed in Figure 6A, genotype effects in the bodyweight indicated a decrease in the groups of 3xTg-AD mice (*p* < 0.05). This effect was mostly due to the notorious weight loss in isolated 3xTg-AD mice, which reached statistical significance as compared to the normal weight of NTg mice (*p* < 0.05). In Figure 6B, the size (weight in mg) and relative size (% vs. bodyweight) of liver, kidneys, and spleen were also recorded as an indirect measure of their physiological/healthy status. Similarly, the bodyweight loss was reflected in the liver, kidneys, and spleen but only reached statistical significance in the excretory (genotype effects, *p* < 0.01) and immune (isolation effect, *p* < 0.001) organs. The analysis of relative weight indicates that the weight loss induced by isolation may refer to a general reduction in somatic size in transgenic animals. Still, the spleen was the most sensitive organ to show the effects of isolation.

Figure 7 shows the weights and biochemical analysis of the levels of tau pathology in the left and right hippocampus, the AD-target area. The size (weight in mg) of L/R hippocampus is depicted in Figure 7A. Differences in L/R hippocampus weight were seen in all the groups (less weight in the right hippocampus) although the hippocampal asymmetry was only attributable to AD-genotype (mean weight; right hippocampus: 12.0 ± 0.57 mg vs. left hippocampus 16.5 ± 0.83 mg, *p* < 0.001). The effects of isolation were seen as a trend of the lower mean weight of the hippocampus. Western blot analysis showed a significant AD-genotype-dependent (*p* < 0.01) increase in phosphorylated tau at Ser396/404 (PHF1) residues in hippocampal lysates of both groups of 3xTg-AD mice as compared with NTg mice (both, *p* < 0.01). The levels of tau did not differ between the right and left hemispheres. Isolation induced in 3xTg-AD mice showed a slight increase in tau, but it did not reach statistical significance.

Meaningful correlation analysis with behavioral variables sensitive to genotype and/or isolation are indicated as insets in Figures 7A,B. The weight of the hippocampus was found to be positively correlated with the activity cage variables *(p* < 0.05), kidney weight (*p* < 0.05), and bodyweight *(p* < 0.05), while it was found to be negatively correlated with the nest building end point at 72 h (*p* < 0.05). The left hemisphere was correlated with the activity test (*p* < 0.05), while the right hippocampus was the one involved with the correlates in body (*p* < 0.05) and kidney *(p* < 0.01) weights, as well as nesting behavior at 24 h (*p* < 0.05). Conversely, the levels of tau pathology were negatively correlated with most time points and total activity developed in the activity-cages and the kidney weight (all, *p* < 0.05).

## Discussion

In the assessment of the long-term effects of preventive/therapeutic interventions, risk factors, and hazards through different periods of our lives, the translational neuroscience approach benefits of the shorter life span of rodents as compared to humans is a key time frame that can be critical in this respect. Like in humans, housing conditions can have a significant impact on animal behavior, and this can also be important in those modeling diseases where social behavior is already impoverished (60). Using these advantages, we can explore in rodents the impact of social isolation or social restrictions due to social distance imposed by COVID-19 and in post-COVID scenarios in the most fragile elder population. Recently, gender differences in COVID-19 found that men are likely to suffer more severe effects of the disease and are more than twice as likely to die (25). On the other hand, it is well-known that dementia is associated with increased mortality, and according to the morbidity/mortality paradox, males show worse survival and deranged neuro-immuno-endocrine system than females despite their decreased bad neuropathological status (2, 3). In the present work, at a translational level, we studied the effects of social isolation in the behavioral, functional, and neuropathological profiles of male 3xTg-AD mice, one of the most used models mimicking the AD shown in humans, at 11 months of age mimicking advanced stages of AD (40), and as compared to age-matched NTg and 3xTg-AD mice housed in standard (grouped) conditions. Fear, anxiety, and agitation are the most common neuropsychiatric symptoms (NPS) associated with dementia that can be studied in mice models of AD (39). They are also called behavioral and psychological symptoms associated with dementia (BPSD) since they also include behavioral disruptive behaviors, they have an early onset, and they worsen with the progression of the disease. Since most behavioral tests assess the animals in behavioral paradigms, the analysis of the actions program (61) allows us to dissect several behavioral domains and constructs including not only the hallmark cognitive dysfunction of AD but also NPS-like behaviors to be recorded (62). For that purpose, a battery of classical unconditioned and ethological tests with the purpose of recording the quantitative and qualitative features of cognitive dysfunction as well as BPSD-like behaviors modeled in 3xTg-AD mice was used (39, 63). The tests evaluated cognition, locomotion/exploration, emotionality, and anxiety-like behaviors in four enclosures differing in the levels of anxiogenic conditions and that, for the purpose of the present work, could resemble or translate into several areas in nursing homes: the mild neophobia response in a cleaned home-cage (Corner test); the direct exposure to an open and illuminated area, which is aversive to nocturnal species like rodents (Open-field); the spontaneous activity and habituation in an activity cage during a long 45-min period (Activity test); and the coping-with-stress strategies and spontaneous alternation in black corridors of a maze resembling burrows (T-maze test). Finally, an ethological nesting behavior was included as a daily life activity involving executive but also protective/thermoregulatory functions. The health status, inferred from their physical status, was monitored by means of the bodyweight and the weight of liver, kidneys, and spleen, organs that show severe complications associated with the COVID-19 scenario (64, 65). Brain pathology of the left and right hippocampus, the hallmark target area in AD, measured by weight and hallmark tau pathology, was performed and correlated with behavior. The main findings refer to an exacerbated (two-fold increase) hyperactivity and emergence of bizarre behaviors in isolated 3xTg-AD mice, worrisome results since agitation is a challenge in the clinical management of dementia and an important cause of caregiver burden. This increase was consistently shown in gross (activity in most of the tests) and fine-motor (thermoregulatory nesting) function. Isolated animals also exhibited re-structured anxiety patterns (negative valence system), emergence of bizarre behaviors, and flight coping-with-stress strategies. Worse risk assessment was dependent on AD-genotype. Bodyweight and kidney weight loss were found in the AD-phenotype and increased with isolation. Isolation-dependent spleen weight loss was observed. Tau pathology in the hippocampus, a target area in AD, was not modified, but asymmetric atrophy of hippocampus, recently described in human patients with dementia and modeled here for the first time in an animal model of AD, was found to increase with isolation. These results are discussed in detail in the next paragraphs.

### Effects of Isolation in the Innate Neophobia

Changes in the innate neophobic response of animals immediately elicited when being held and put in a new place, before they can recognize it as novel or familiar, is one of the most sensitive ethological behaviors of the early phenotype in the 3xTg-AD mice modeling AD (39, 41, 55, 66, 67). Here, we benefit from the use of a clean standard home-cage to transport the animal to the area where the open field will be performed, to assess this immediate fearful response. As shown, both horizontal and vertical components confirmed that house-grouped 3xTg-AD mice exhibited increased levels of fear in this natural neophobia response as compared to age-matched NTg mice. This was shown as a two-fold decrease in horizontal and vertical activities and a longer time to elicit the first rearing. The delay in vertical exploratory activity was also present in isolated 3xTg-AD animals, indicating an increased fearful response. However, differences with NTg counterparts were lost since the coping strategy exhibited was a fight-to-flight behavior that counteracted the low activity levels due to the AD-genotype. This was similar to the psychostimulant effect that our laboratory has shown in this animal model after chronic treatment with caffeine (68).

### Effects of Isolation in the BPSD-Like and Cognition in the Open Field and Activity Tests

When confronting an open and illuminated environment such as the open field, both group-housed mice exhibited a poor exploratory activity pattern. This result was confirmed during the first 5 min of the activity test, where equal performances were also observed in NTg and 3xTg-AD mice. In this other test, which did not reach statistical significance, the subsequent analysis of the long-term habituation in the activity cages showed a worse (lower) pattern in the 3xTg-AD mice. Our laboratory has previously described the convergence of profiles in the context of poor aging and related physical and cognitive declines in NTg mice as part of the complexity of age-related scenarios and heterogeneity among all populations, including wild-type mice (not just 3xTg-AD mice) (69). Interestingly, these smooth patterns in middle-aged mice housed in standard conditions also emphasized the impact of isolation shown as prominent levels of both horizontal and vertical activities. This effect was more noticeable in the horizontal locomotor component of activity as they were sustained from the very first minute of the test, in agreement with the flight-to-fight response shown also in the corner test and all the subsequent behavioral tests. As in the other two groups, the levels of vertical exploratory activity of isolated 3xTg-AD mice were mostly null at the beginning of the test, in agreement with the immediate fear response in an anxiogenic open and illuminated enclosure. However, according to the action program described by Lát (61), as soon as this initial fear was managed, the level of vertical exploration rose and progressively increased during the test until its end. Isolation not only increased the levels of standard locomotor and exploratory behaviors but also elicited the emergence of bizarre actions not shown in wild-type mice. Here, the presence of vertical activity without wall support, indicative of an escape behavior (55), was scarcely seen in 3xTg-AD mice (only one animal, late-onset) but was exhibited early in half of the 3xTg-AD mice housed in isolation conditions. This result reinforces the above-mentioned increased active fearful response induced by isolation.

In contrast to the open field test, the low anxiogenic levels of activity cages facilitate the expression of hyperactive patterns as already shown in our laboratory in 12-month-old female 3xTg-AD mice (39). Therefore, the activity levels recorded here, with a maximum of 10 m in group-housed NTg and 3xTg-AD mice but 20 m in isolated 3xTg-AD mice, emphasized the noticeable hyperactivity pattern induced by isolation of individuals. Thus, the results in spontaneous locomotor activity not only confirmed the hyperactive fearful response of isolated 3xTg-AD mice already shown in the previous tests (corner test for neophobia and the 5 min of the open field test) but also demonstrated that the effect of isolation was sustained on time, despite habituation to the enclosure. Differences were maintained statistically significant during 40 of the 45 min duration of the test. This is important because habituation is considered the simplest form of learning and memory (70, 71). In fact, the results would also be in agreement with the psychostimulant effects induced by chronic treatment with a low dose of caffeine in this animal model of AD as measured in a spatial learning cognitive task (68).

### Effects of Isolation in the BPSD-Like and Cognition in the T-Maze

The spontaneous alternation in the T-shaped maze with black and narrow corridors resembling burrows is a paradigm mostly used to evaluate spatial working memory in a naturalistic manner. More recently, some authors have dissected other cognitive aspects (72). The test also involves emotionality/anxiety and exploratory activity as we have shown extensively, in both male and female mice, from young to old age under healthy and diverse neuropathological conditions (39, 62, 63, 66, 73). This neuropsychiatric component was also pointed out here by the correlation analysis. Thus, the T-maze ethogram correlated with that shown in the open-field test, the horizontal and vertical activities in the first minute where a fearful response is elicited, and the total horizontal and vertical activities developed.

Interestingly, the latency to cross the intersection of the maze allows us to assess the coping-with-stress strategies of animals. Alterations in this parameter, as compared to the standard wild-type performance to cope, have been found to correlate with a worse neuro-immunoendocrine function, indicators of accelerated aging and premature death in mice (74). Although no significant differences were observed in the number of errors in the spontaneous alternation behavior, most 3xTg-AD mice revisited areas of the maze previously explored. The 3xTg-AD mice and those isolated exhibited opposite performances since the latency to reach the intersection and the time to complete the maze were delayed or fastened, respectively, in a statistically significant manner. In previous studies, we have shown that young 3xTg-AD mice mimicking prodromal stages of disease exhibit a fast-speed performance to reach the intersection in comparison with age-matched NTg mice, in a typical flight behavior in the fight-or-flight strategy. At old or middle ages, like those of the animals used here, a slow performance involving petrification is the most common behavioral response (75). In the present work, the fast pattern in isolated animals, resembling that of chronically psycho-stimulated 3xTg-AD mice (68), suggests that the hyperactive pattern also involves a switch in their coping-with-stress strategies. The impaired decision-making process is shown as a delay to cross the intersection, a function that depends on prefrontal cortical areas, and was not modified by isolation. We have previously reported similar delays in the decision to move from one side to another in the dark-light box test, suggesting that the AD genotype models differences in risk assessment (39, 62, 63, 66). Sensorial tactile stimulation known to modulate cognitive and anxiety-like behaviors hampered this phenomenon in the 3xTg-AD mice (76, 77) and infantile cerebral palsy model (78). Impairment in working memory in other spatial tasks has also been reported in APP/PS1 and Tg2576 mice after social isolation (79, 80). The conspicuous neuropsychiatric-like profile of 3xTg-AD mice models the complexity of the advanced stages of the disease where behavioral alterations disrupt cognitive function and cause the most important burden of disease for the patients and caregivers. In fact, in addition to cognitive aspects, pharmacological management of dementia targets agitation, irritability, aggressiveness, disorientation, and wandering among other neuropsychiatric symptomatology. In this respect, our most recent translational study on the pharmacological treatment of these endophenotypes also found efficiency to reach the goals in this T-maze task being compromised in middle-aged male 3xTg-AD mice, a poor performance that was slightly affected by long-term treatment with the antipsychotic risperidone (81).

### Effects of Isolation in the Daily Life Activity

Deterioration in daily life activities and executive functions are early functional signs in patients with dementia. They worsen with the progress of disease and demand important caregiver attention (82). On the other hand, age-related deficits in thermoregulation are more pronounced in AD patients (83). The impact of isolation in these questions was addressed, at the translational level, by means of nesting behavior, an innate ethological inference to assess well-being and daily life activity in normal mice and models for neuropsychiatric diseases (58, 59, 84). Conversely, nesting material is considered a tool for the improvement of the animal's welfare. Nesting behavior is observed in the context of maternal affiliative behaviors, facilitating family structures, maternal interaction, and protecting the offspring from outsiders and predators. However, nesting behavior also extends to males and non-pregnant females driven by protection and thermoregulation needs. We first described deficiencies of 3xTg-AD mice in this instrumental task involving executive functions and as dependent on the protocol and nesting material. As we demonstrated, a 3-day assessment with paper towel instead of standard protocols (using cotton, assessing only at 24 h) revealed genotype-, gender- and age-dependent differences in 3xTg-AD mice under different social settings (58). In the present work, the mean nesting scores and 3-day patterns for NTg and 3xTg-AD mice are in agreement with those observed in 12-month-old NTg and 3xTg-AD males (isolated during the 3 days of nest-building assessment) as reported in our previous work (58). Here, we demonstrate that long-term isolation of 3xTg-AD mice not only resulted in hyperactive locomotor patterns but also higher nest-building scores and smaller nests. This can be understood in the context of “thermoregulatory nesting,” a behavioral construct demonstrated by geographic cline in nest-building behavior (85). Thus, the lower core body temperature of aged 3xTg-AD mice (86), the worse thermoregulation shown also in other models (87), and the loss of group-sleeping behavior in isolated 3xTg-AD animals may have enhanced the utility of nests to cover increased metabolic, thermoregulation, and protective needs. Regarding their hyperactive phenotype, it is interesting to note that several studies have identified a negative genetic correlation between thermoregulatory nesting and wheel-running activity with increased wheel running in mice selected to build small nests (88, 89). Inverse relation of nesting with body mass and litter size (85) and body temperature (90) has also been demonstrated and would be in agreement with the trend to a lower bodyweight of isolated 3xTg-AD mice found here. Overall, the present results point out that isolation induces increases in both gross (hyperactivity) and fine (nesting) motor function. Therefore, maximal aerobic capacity enabling such sustained activities, resting metabolic rates, thermoregulatory abilities, and energy requirements are key aspects to take into account in the isolation scenarios (89).

### Effects of Isolation in the Physical Status

Overweight, a characteristic of the Spanish colony of 3xTg-AD mice (63), was not found in this particular experimental set. However, a trend of reduced weight was found in isolated animals resembling the effects we described previously in male 3xTg-AD mice receiving long-term treatment with caffeine (68). In that work, we measured the psychostimulant effects of caffeine in a whole light/dark cycle. We observed that hyperactivity in their diurnal sleeping period was followed by enhanced expression of hyperactivity in their active nocturnal activity period, which could explain their weight loss. In other words, we have also reported that 3xTg-AD are more sensitive than NTg mice to weight loss under the stressful environment of a forced treadmill exercise (63) but not voluntary wheel running (66). In those experiments, the decrease in weight was more evident in the females, both because of their higher corticosterone response and their lower basal weight than males. Therefore, the bodyweight decrease found in the present work in isolated males may be of functional significance. As mentioned previously, the results of weight and relative weight suggest that isolation may induce a general weight loss or reduction of somatic size in 3xTg-AD mice. Separation of mice increases metabolic demands and has been proposed as a new and easy-to-perform animal model for weight loss (91). Sex differences in the psychobiological effects in Swiss kumming mice undergoing 1–4 months social isolation have also been demonstrated (92). There, both isolated males and females showed less bodyweight gain; however, male mice were more affected by isolation.

### Effects of Isolation in the Metabolic, Excretory, and Peripheral Immunity Organs

Although brain oxidative stress in AD is accepted, the contribution of the disease to peripheral oxidative redox state has been little studied. In this regard, we reported peripheral alterations (41, 63) in early oxidative stress status in male and female 3xTg-AD mice, with a decrease in antioxidant defenses and an increase in xanthine oxidase activity in most peripheral tissues, among them the liver, kidney, and spleen studied here (93, 94). Interestingly, these genotype differences were more exacerbated in male than in female mice. In the present and other works, we used the size and relative size of liver, kidneys, and spleen as an indirect measure of their physiological status as we have proposed that these parameters can be a tool for sonographic monitoring of patients (41, 75). Other laboratories have also confirmed in this animal model this liver and spleen dysfunction reflected as hepato- and splenomegaly (95, 96) and fatty liver pathology (97). Consistent associations of liver function with AD patients has been recently described, indicating metabolic disturbances (98). In the current pandemic, persistent inflammatory syndrome is an important negative prognostic indicator for survival. A relation with COVID-19 and liver dysfunction has been reported (65), and metabolic-associated fatty liver disease has been related to a worse evolution of COVID-19 (99).

Since our first report (41) proposing sex-specific immuno-endocrine aging in old 3xTg-AD mice (worse in males), we have consistently reported thymus involution and splenomegaly as characteristic of the impairment of the neuro-immune-endocrine system in this animal model (100). The easy measurement of their weight and relative weight (organometrics), with clinical translation, can be used as early indicators of peripheral immunological system aging (75, 93, 100), as also confirmed by other laboratories (96). We have shown previously that the alterations in the brain redox state (66, 67) develop in parallel to the impaired splenic redox state, measured as an increased xanthine oxidase activity and lower total glutathione levels and glutathione peroxidase and reductase activities, as well as in terms of pro-inflammatory (IL-10 decreased)/anti-inflammatory (IL-1beta and TNF-alpha increased) balance in leucocytes in male and female 3xTg-AD mice since the prodromal stages of the disease (93, 101). In the present work, weight and relative weight of spleen were found reduced in isolated 3xTg-AD mice, mimicking again the effect of long-term stimulant effects of caffeine (68). The increase in chronic physiological stress induced by long-term isolation may also support these results since elevated levels of stress have been related to a decreased spleen mass (102, 103) and models of physical stress combined with social isolation show a worsening of the immuno-endocrine system (104).

With regard to the kidney, acute renal impairment complicating viral infection was already observed in some patients affected by SARS-CoV (105). Although AKI was reported as uncommon and likely to be related to multi-organ failure rather than the viral tropism in the kidney, it was recognized as carrying a risk of high mortality. The prevalence of kidney disease in patients with COVID-19 on admission and the development of acute kidney injury during hospitalization are high and are also associated with in-hospital mortality (64). As a *Kidney International* editorial noted, early risk identification, rapid and effective treatments, and avoidance of nephrotoxins may help provide a better prognosis of patients with COVID-19. A case report also describes the rapid development of collapsing glomerulopathy, which further deteriorated despite the improvement of respiratory symptoms. In this sense, the linkage between chronic kidney disease and AD may suggest a higher vulnerability in AD patients in the COVID-19 pandemic (95). At the translational level, we have preliminary observations on severe glomerular affectation of kidneys in 3xTg-AD mice. That also would be in agreement with the correlation analysis that, in the present work, found a relation between weight loss of the hippocampus and its relative levels of tau with the kidneys' weight loss. Therefore, the AD-genotype effects found in the present work in the weight loss in the kidneys worsen by isolation and may suggest clinical implications.

### Effects of Isolation in the Right/Left Brain Asymmetry

Several psychiatric disorders and psychological conditions associated with stress and trauma, such as PTSD (106) and major depressive disorder (107), are associated with a reduction in hippocampal volume and alterations in hippocampal memory function as a result of the key role of the hippocampus in the regulation of the hypothalamic-pituitary-adrenocortical axis in acute and chronic stress (108). In addition, brain hemispheric left–right asymmetry and lateralization is known to exist at several anatomical levels in the brain, and hippocampal asymmetry has been suggested to indicate lateralized differences in vulnerability to trauma (106). A meta-analysis also shows that hippocampal atrophy was associated with trauma exposure independent of PTSD diagnosis, PTSD-induced additional hippocampal reduction, with the right hippocampus being smaller in the PTSD group than the trauma-exposed group without PTSD (109). Also, in the context of the present work, the whole-brain analysis has revealed increased neuroanatomical asymmetries in dementia for hippocampus and amygdala (110). At the translational level, recent studies in rodents have explored left–right hippocampal asymmetry, providing further evidence at the neurochemical and molecular levels with regard to the glutamatergic system (111). The lateralization of the stress regulatory system has also been shown, with long-term isolation stress in rats producing right–left asymmetry of the hippocampus norepinephrine and changes in catecholamine synthesis and reuptake (112). At the functional level, hippocampal asymmetry is important for the acquisition of spatial reference memory and retention of working memory (113) as wells as for certain characteristics of non-spatial learning (111). Here, in the present work, our results show that in all the animals, the weight of the hippocampus from the right hemisphere was lower than that of the left hemisphere and that this asymmetry was increased in the animals with AD-genotype, reaching the maximum statistical significance. To our knowledge, this is the first time that increased asymmetry in the hippocampus of an animal model of AD is demonstrated, thus modeling the above-mentioned asymmetry found in AD human patients. This modeling will be useful for the translational development and assessment of preventive/therapeutic interventions and those of the risk factors and hazards, as well as monitoring disease progression. In this respect, it already indicates that isolation induces an increase of one degree of magnitude in the statistical significance of the left–right differences since in the 3xTg-AD mice housed in standard conditions the left–right hippocampal asymmetry reached *p* < 0.05 while in isolated 3xTg-AD mice the significance reached the an order of magnitude of *p* < 0.01. Further quantitative and qualitative analysis of regional neuronal density in CA1, CA2, and ventral hippocampus to quantify distinct neuron depletion may reveal a regionally gradient neuronal loss, specific also for the hippocampus of each hemisphere. Interestingly, in the present work, the weight of the left hippocampus was maintained constant, while the right hippocampus decreased with genotype and with isolation, suggesting hemisphere-dependent cellular vulnerability.

### Behavioral Correlates of Pathological Status

Considering the three groups, the behavioral correlates of neuropathology indicated that the levels of tau were negatively correlated with most time points and total activity developed in the activity-cages and the weight of the kidneys. Conversely, the total weight of the hippocampus was found positively correlated with the kidneys and bodyweights, as discussed previously, as well as the performance in the activity, but negatively correlated with the nest building. Considering each hemisphere, lateralization was found since the left hippocampus correlated with the activity, while the right hippocampus positively correlated with bodyweight and kidney weights, and negatively with nesting behavior. However, the activity patterns of the two 3xTg-mice groups were allocated in two opposed positions concerning the activity shown by NTg mice (low in 3xTg-AD mice, high in isolated 3xTg-AD mice): the selective atrophy in the right hippocampus in AD-genotypes and its enhancement by isolation. Altogether, the correlation analysis may show the overall relationship between weight and activity but cannot be directly related to the hippocampal asymmetry found in the AD-genotype or the loss of weight of the right hippocampus and the enhancement of these aspects by isolation.

### Social Isolation in Other Animal Models of Alzheimer's Disease

Finally, studies in Tg2576, APP/PS1, 5xFAD mice have reported effects of social isolation in the AD pathology as related to an increase in Aβ peptide (48, 80, 114). However, the work by Pietropaolo et al. (50) assessing the impact of isolation in the 3xTg-AD mice from post weaning (postnatal day 21) until the onset of disease (6 months of age) found no significant changes. Our present report, in a complementary manner, studied the impact of a similar period of isolation on advanced stages of the disease, in naturally occurring scenarios (disruptive behaviors or death of partners). The biochemical analysis of these samples confirmed the increased relative levels of phosphorylated tau protein in the hippocampus of 3xTg-AD mice as measured by PHF1, with a significant genotype effect but no left–right differences. Isolation induced a slight increase in the AD-pathology but did not reach the statistical significance.

### Conclusions

In summary, the present study shows that single-housed 3xTg-AD for AD showed a prominent hyperactive pattern as shown in gross motor function in all the tests, the emergence of bizarre behaviors, and intensified nesting behavior (fine-motor function), probably responding to increased demands of protection and thermoregulation induced by isolation in an already AD-frail scenario. Anxiogenic patterns were broken and coping-with-stress strategies were changed (from freeze) to flight behavior in isolated 3xTg-AD male. Although isolation did not exacerbate the hallmark tau pathology in the hippocampus, we could demonstrate, for the first time, hippocampal atrophy and left–right asymmetry and lateralization (behavioral correlates). Hippocampal atrophy was selective of the right hemisphere and related to bodyweight and kidney weight loss and (negatively) nesting behavior. Isolation-induced weight loss was observed in peripheral immune organs (spleen). Despite limitations of translating animal models into human application, these results point at isolation having an impact on aspects that increase the intrinsically AD-associated deficits and vulnerability. It would be interesting to perform a translational approach of these preliminary findings in a clinical sample of AD patients to study the impact of COVID-19–related social isolation from a psychological and neuroimaging (fMRI) point of view. The present results help make us aware of the severe impact of the new physical distancing measures not only on those expected in cognition but also on the expression of BPSD. More importantly, the results point to the worsening of the AD-brain hippocampal asymmetry. These results from translational neuroscience research models provide guidance about the complexity of the new scenarios. This will be important when considering the best preventive/therapeutic strategies in times of coronavirus and the post–COVID-19 pandemic. From our perspective, there is a strong need for non-pharmacological interventions for managing BPSD as those provided by the last international consensus (i.e., caregiver training, environmental adaptations, music therapy, and other person-centered care and activities) before any pharmacological approaches (8, 115, 116). The coordinating role of different health system actors (geriatricians, nurses, psychologists, occupational therapists, physiotherapists) will be critical for the implementation and success of these interventions (117). Thus, the present work also highlights the relevance of personalized-based interventions tailored to the heterogeneous and complex clinical profile of the individuals with dementia as well as the effects that the worsening of some symptoms have on the caregiver burden.

## Data Availability Statement

The original contributions presented in the study are included in the article/supplementary material, further inquiries can be directed to the corresponding author/s.

## Ethics Statement

The animal study was reviewed and approved by CEEAH Universitat Autònoma de Barcelona & Generalitat de Catalunya.

## Author Contributions

LG-L: conceptualization. AM: performance, analysis of behavior, and illustrations. Both authors: equally contributed to the scientific discussions, writing, and approval of the manuscript.

## Conflict of Interest

The authors declare that the research was conducted in the absence of any commercial or financial relationships that could be construed as a potential conflict of interest.
